# Effects of Aneurysmal Subarachnoid Hemorrhage in Patients Without In-Hospital Infection on FABP-I, LBP, and sCD-14

**DOI:** 10.3390/ijms26020485

**Published:** 2025-01-08

**Authors:** Brigitta Orban, Diana Simon, Szabina Erdo-Bonyar, Timea Berki, Tihamer Molnar, Laszlo Zavori, Attila Schwarcz, Zoltan Peterfi, Peter Csecsei

**Affiliations:** 1 Department of Neurosurgery, Medical School, University of Pecs, 7622 Pecs, Hungary; orbanbrigi8@gmail.com (B.O.); schwarcz.attila@pte.hu (A.S.); csecseipeti@yahoo.com (P.C.); 2 Department of Immunology and Biotechnology, Medical School, University of Pecs, 7622 Pecs, Hungary; simon.diana@pte.hu (D.S.); erdo-bonyar.szabina@pte.hu (S.E.-B.); berki.timea@pte.hu (T.B.); 3 Department of Anaesthesiology and Intensive Care, Medical School, University of Pecs, 7622 Pecs, Hungary; 4 Emergency Department, Saudi German Hospital, Dubai 61313, United Arab Emirates; zavori.laszlo@gmail.com; 5 1st Department of Medicine, Medical School, University of Pecs, 7622 Pecs, Hungary; peterfi.zoltan@pte.hu

**Keywords:** aneurysmal subarachnoid hemorrhage, intestinal fatty acid binding protein (FABP-I), lipopolysaccharide binding protein (LBP), sCD-14, leaky gut syndrome, inflammation, intestinal permeability, endotoxin

## Abstract

Aneurysmal subarachnoid hemorrhage (aSAH) is a serious condition complicated by delayed cerebral ischemia (DCI), where inflammation plays a key role. Although altered gut permeability is noted in other conditions, its significance in aSAH remains unclear. Fatty acid-binding protein (FABP-I), lipopolysaccharide-binding protein (LBP), and soluble CD-14 (sCD-14) are established markers of barrier dysfunction. This study investigates gut permeability marker changes in early and late aSAH phases. The study included 177 aSAH patients and 100 controls. Serum samples were collected on days 1 (D1) and 9 (D9) after ictus. FABP-I, LBP, and sCD-14 levels were measured via ELISA, and clinical data were recorded. Outcomes were assessed using the 90-day modified Rankin scale (mRS 0–3 = favorable outcome). Serum FABP-I was significantly lower in aSAH patients (*p* < 0.05), while LBP and sCD-14 were higher (*p* < 0.001) compared to controls. FABP-I did not differ between outcome groups, but LBP and sCD-14 were significantly elevated in unfavorable outcomes (*p* < 0.001). These markers differed in patients without in-hospital infection, with higher levels noted in DCI patients during the later phase (*p* < 0.05). In aSAH patients without infection, differences in LBP and sCD-14 levels between outcome groups suggest potential endotoxin release from microbial systems, contributing to neuroinflammation and influencing outcomes.

## 1. Introduction

Aneurysmal subarachnoid hemorrhage (aSAH) is a severe cerebrovascular event, representing 5% of all strokes. The spontaneous rupture of an intracranial aneurysm is the primary cause of aSAH and places significant strain on healthcare resources, as it triggers a cascade of events that result in organ dysfunction [1]. Hypertension, smoking, and the use of cocaine or alcohol are factors that significantly increase the risk of cerebral aneurysm rupture [2]. It has a high mortality rate of 25% and leaves 66% of survivors with lasting disabilities [3]. Despite a decline in age-standardized rates over time, the burden of subarachnoid hemorrhage remains substantial [4]. Studies have reported higher incidence rates in countries like Japan and Finland, with rates of 22.7 and 19.7 per 100,000 person-years, respectively. In contrast, regions such as South and Central America have reported lower incidence rates, averaging around 4.2 per 100,000 person-years [5]. Careful management in the intensive care unit (ICU) has greatly enhanced the prognosis of aSAH [6]. Increased intestinal permeability is a serious issue among patients treated in the ICU [7]. In critically ill patients, the rise in intestinal permeability (″leaky gut″) is linked to a higher incidence of bacterial and toxin translocation from the intestinal lumen into the systemic circulation [8,9].

Several underlying causes of leaky gut are known, such as intestinal hypoperfusion, enterocyte apoptosis, systemic cytokine storm, gut dysbiosis [10], immune-complex deposition in the gut, adverse effects of medications [11], or stress hormone-induced immune alteration involving the autonomic nervous system [12]. Increased intestinal permeability can have severe consequences due to its disruption of the intestinal barrier such as bacteremia (presence of bacteria, bacterial fragments, and toxins in the blood) which contributes to the development of systemic inflammatory response syndrome [9], increased susceptibility to secondary infections [13], and altered nutrient absorption [14]. Furthermore, leaky gut precedes and boosts the development of certain neurological diseases [15]. Various circulating biomarkers have been evaluated and validated for assessing intestinal barrier integrity and bacterial translocation. Intestinal fatty acid binding protein (FABP-I), also known as FABP2, is a protein expressed in the intestine, with the highest expression observed in the jejunum [16]. Its functions include the absorption and metabolism of intestinal fatty acids and the regulation of triglyceride levels [17]. The role of FABP-I as an indicator of barrier integrity is well-established [18,19,20,21,22]. Lipopolysaccharide binding protein (LBP) is a soluble acute-phase protein that binds to bacterial lipopolysaccharide (LPS), initiating immune responses by presenting the LPS to key cell surface pattern recognition receptors, such as CD14 and TLR4 [23]. LPS exposure triggers LBP production. Plasma LBP is considered a more reliable biomarker for detecting plasma LPS levels than LPS itself, due to LPS’s short half-life [24]. LBP might be a valuable biomarker for the assessment of intestinal permeability in adults [25,26,27]. The LPS–LBP complex subsequently interacts with CD14, initiating a cascade of inflammatory responses [28].

sCD14 is a receptor molecule primarily produced by macrophages and hepatocytes as part of the innate immune response to LPS [29,30]. Measurements of sCD14 in blood, in conjunction with proteins which are released into circulation when gastrointestinal (GI) tract epithelial cells are damaged (FABP-I), are good indicators of systemic microbial translocation [31]. Further support for the role of sCD14 as a marker of gut barrier dysfunction comes from studies conducted in HIV research [32,33] and immunology [34].

The measurements of FABP-I, LBP, and sCD-14 do not require complex methods and their concentrations can be accurately detected in peripheral blood [35], so measuring them together can provide reliable information regarding the intestinal permeability status.

Thus, the main goal of our study is to determine the serum levels of these markers in patients with aSAH during the early and late phases in order to assess changes in intestinal permeability, and to identify any correlations with 3-month favorable and unfavorable outcomes (as primary objective) and with delayed cerebral ischemia (secondary objective).

## 2. Results

### 2.1. Patients Characteristics

A total of 206 patients with first-ever aSAH underwent initial evaluation. Among them, 24 patients were excluded because of the following reasons: severe underlying systemic diseases (11), aneurysm rebleeding (2), severe kidney failure (2), loss of follow-up (5), and unavailable biomarker measurements (9). Finally, 177 subjects with aSAH met the eligibility criteria and completed the entire study protocol (see File S1 for patient recruitment flowchart). Among these 177 patients, 128 (72%) were females, 56 (32%) smoked cigarettes. The mean age was 57.8 years (SD, 12 years). A total of 100 healthy controls with unruptured intracranial aneurysm were recruited. All patients included in the study underwent endovascular treatment; no patients who received open surgery were included in the study. Among these 100 controls, 71 were females and their mean age was 59.6. years (SD, 12 years). There was no significant difference in age or the proportion of females between the aSAH patient and control groups. Table 1 provides detailed aSAH group characteristics.

### 2.2. Serum Level of FABP-I, LBP and sCD-14 in aSAH Patients and Controls

**Figure 1:**
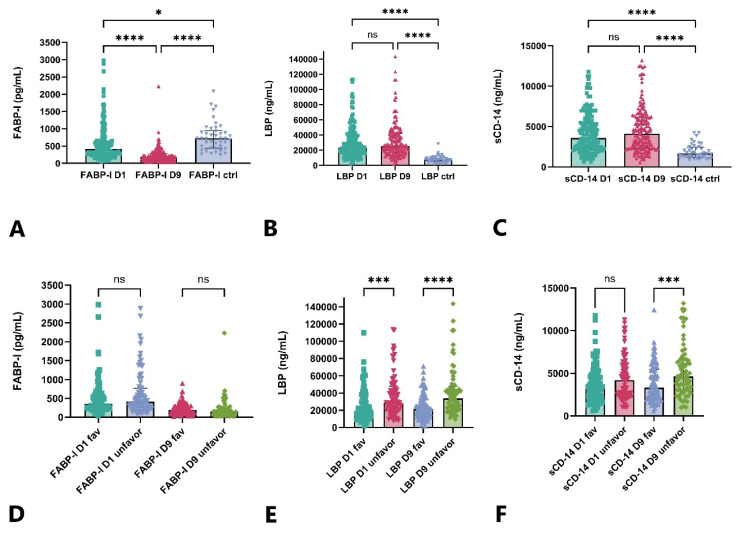
Serum level of FABP-I, LBP and sCD-14 in aSAH patients versus controls (**A**–**C**) and in different outcome groups (**D**–**F**). FABP-I, fatty acid-binding protein-intestinal, LBP, lipopolysaccharide-binding protein, D1, sampling time 24 h after ictus, D9, sampling time 9 days after ictus, fav, favorable (modified Rankin score 0–3) outcome, unfavor, unfavorable (modified Rankin score 4–6) outcome, ns, non-significant, * denotes *p* < 0.05, *** denotes *p* < 0.001, **** denotes *p* < 0.0001, number of control subjects: 100, number of patients with favorable outcome: 94, number of patients with unfavorable outcome: 83.

The serum FABP-I level was significantly lower in the aSAH patient group (FABP-I levels of D1 and D9 samples) compared to the control group (*p* < 0.05). Within the patient group, the FABP-I levels in the late samples (D9) were significantly lower (*p* < 0.001) compared to the early samples (D1), Figure 1A. In the case of LBP, we observed significantly higher (*p* < 0.001) serum levels in the patient group compared to the control group; however, no difference was found between the levels in the early and late samples, Figure 1B. We observed similar results in the examination of serum sCD-14 levels, with significantly higher (*p* < 0.001) serum sCD-14 levels in the aSAH group compared to the control group. However, there was no substantial difference between the D1 and D9 serum sCD-14 levels. The exact values of the markers measured in the patients and controls are included in File S2.

### 2.3. Serum Levels of FABP-I, LBP, and sCD-14 According to Outcome Groups and the Occurrence of DCI

**Figure 2:**
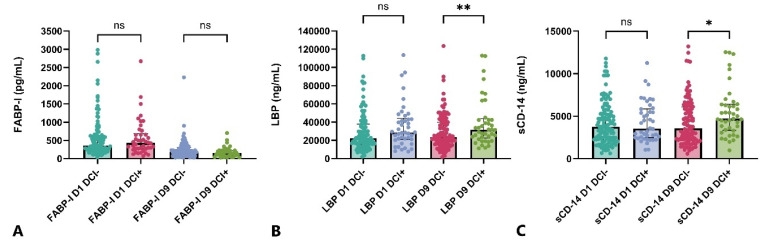
Serum level of FABP-I (**A**), LBP (**B**) and sCD-14 (**C**) in patients with and without delayed cerebral ischemia. DCI delayed cerebral ischemia, *+*, patients with DCI, −, patients without DCI, FABP-I, fatty acid-binding protein-intestinal, LBP, lipopolysaccharide-binding protein, ns, non-significant, * denotes *p* < 0.05, ** denotes *p* < 0.01, number of patients with DCI: 47, number of patients without DCI: 130.

In the analysis of FABP-I levels, no differences were observed in the serum levels of either the early or late samples between the two outcome groups (Figure 1D). In the group with unfavorable outcomes, significantly higher LBP serum levels were observed compared to the favorable outcome group at both measurement time points (D1, D9) (Figure 1E). A significant difference in serum sCD-14 levels between the two outcome groups was observed only at the D9 sampling time point, while no difference was detected in the early sample taken 24 h after the ictus (Figure 1F). None of the three examined metabolites showed any correlation in the early phase (D1) of aSAH with the occurrence of delayed cerebral ischemia. In contrast, both LBP and sCD-14 reached significantly higher serum levels in the DCI subgroup in the later phase (D9) (Figure 2).

### 2.4. Serum Levels of FABP-I, LBP, and sCD-14 and In-Hospital Infection

**Figure 3:**
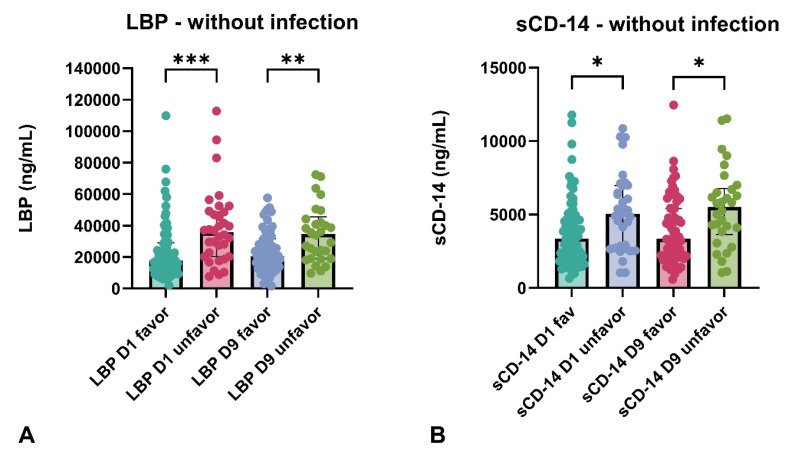
Serum levels of LBP and sCD-14 in aSAH patients without in-hospital infection according to 3-month outcome. The serum LBP level in the aSAH group without infection (**A**), the serum sCD-14 level in the aSAH group without infection (**B**). D1, sampling time 24 h after ictus, D9, sampling time 9 days after ictus, favor, favorable (modified Rankin score 0–3) outcome, unfavor, unfavorable (modified Rankin score 4–6) outcome, * denotes *p* < 0.05, ** denotes *p* < 0.01, *** denotes *p* < 0.001, number of patients in each group, favorable outcome: *n* = 61, unfavorable outcome: *n* = 55.

We also examined the serum levels of LBP in the context of hospital-acquired infections and found that in patients who did not develop an infection (*n* = 116) during hospitalization (median [IQR] serum level of CRP during hospitalization: 5.7 [2–12]), LBP reached significantly higher levels in the unfavorable outcome group compared to those with favorable outcomes at both measurement time points (Figure 3A). We found a similar but weaker correlation in the case of sCD-14 as well (Figure 3B). In patients who developed an infection (*n* = 61) during hospitalization (median [IQR] serum level of CRP during hospitalization: 73.4 [53–112]), we did not observe any difference in serum levels of LBP or sCD-14 between the favorable and unfavorable outcome groups at any measurement time point. In the group without infection (*n* = 116), we performed a correlation analysis between serum CRP levels and early (D1) and late (D9) LBP and sCD-14 values. We did not observe a significant correlation for any marker in either the early or late phase.

**Figure 4:**
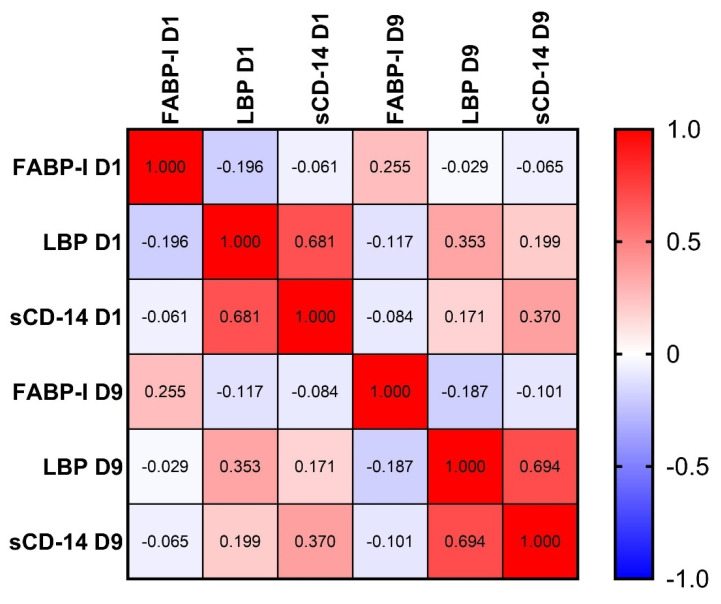
Heat map showing correlation matrix of FABP-I, LBP and sCD-14. In heatmap, red indicates positive, while blue indicates negative correlation. The darker the color, the stronger the correlation. The Spearman rank correlation test was used. A *p*-value < 0.05 was accepted as significant. *p* > 0.10 are presented in white squares. FABP-I, fatty acid-binding protein-intestinal, LBP, lipopolysaccharide-binding protein, D1, sampling time 24 h after ictus, D9, sampling time 9 days after ictus.

In addition, very strong positive correlations were observed between LBP and sCD-14 in both the early (D1) and late (D9) phases of aSAH (Figure 4). In addition, we did not observe any significant correlation among the three metabolites and factors such as age, gender, smoking, diabetes, and hypertension.

## 3. Discussion

In this study, we found the following key results: (i) the serum levels of LBP and sCD-14 were significantly higher in the aSAH cohort compared to the unruptured control group, while the serum level of FABP-I was significantly lower in the patient group compared to control, (ii) the serum level of FABP-I did not differ significantly, whereas LBP and sCD-14 levels (specifically in the D9 samples) were significantly different between the outcome groups, (iii) LBP and sCD-14 showed a difference between the favorable and unfavorable outcome groups in patients without in-hospital infections, while (iv) LBP and sCD-14 were significantly higher in the DCI group during the later phase, (v) LBP and sCD-14 exhibited a strong positive correlation in both the early and late phases of aSAH.

First, we found that FABP-I was significantly lower in the aSAH cohort than in the control group, which was contrary to our expectations. Moreover, the decrease between the early and late phase values within the patient group was also significant. This result is unexpected, considering that several studies involving patients with ischemic or inflammatory gastrointestinal conditions [36,37,38], or those who had experienced traumatic brain injury [39], reported higher levels compared to the healthy control group. Similarly, elevated serum FABP-I levels have also been observed in certain psychiatric disorders [40,41] and in ischemic stroke [42]. Our results may have several explanations.

Theoretically, a reduced level of FABP-I may reflect diminished enterocyte mass or altered intestinal function. In our previous study [43], we found that in aSAH patients, metabolism shifts towards ketosis and alternative energy sources in both the early and late phases, even with adequate enteral nutrition, particularly in patients with poor outcomes. Other studies also have highlighted that the nutritional status of aSAH patients, both at the time of admission and throughout their hospitalization, plays a crucial role in affecting their clinical outcomes [44,45,46,47]. It is well-established that energy expenditure significantly increases in patients with aSAH following the onset of the condition [47,48], but we also know from an international, multicenter ICU study that aSAH patients were routinely underfed, receiving less than 60% of both total caloric and protein intake [49]. Additionally, Hernandez et al. found that a short period of enteral fasting was associated with significant duodenal mucosal atrophy and abnormal gut permeability in critically ill patients [50]. Based on this, we can assume that inadequate intake of nutrients can impair intestinal health and reduce enterocyte proliferation, and the early malnutrition of aSAH patients may lead to decreased synthesis of I-FABP due to a reduced number of functional enterocytes.

In our study, we also found that LBP and sCD14 levels were significantly higher in the poor outcome group compared to the favorable outcome group. LBP is a soluble protein that binds to LPS and delivers it to CD14, triggering the activation of target cells via toll-like receptors (TLRs), which play a crucial role in regulating innate immunity [51,52]. CD14 also exists in a soluble form (sCD14) [53]. LBP levels in the serum reach their highest shortly after the onset of bacteremia or endotoxemia and remain elevated for up to 72 h [54]. Once in the bloodstream, LBP binds to LPS, facilitating its interaction with CD14 receptors [55,56]. Membrane-bound CD14 is linked to TLR4, which transmits the signal from the CD14-bound LPS to the cell’s nucleus, initiating a cascade that leads to the release of inflammatory cytokines [57]. The LBP level has been proposed as a clinical indicator of “active endotoxemia” [58,59]. Elevated LBP levels may indicate exposure to bacterial components, particularly LPS, and could serve as a predictor of disease progression and unfavorable outcomes [54,60,61,62]. The increase in LBP concentration has been observed in various metabolic diseases, which are linked to changes in gut permeability and alterations in the gut microbiome [63]. An increase in plasma endotoxin activity was associated with higher levels of LBP and sCD14. Elevated concentrations of both LBP and sCD14 can inhibit the bioactivity of LPS both in vitro and in vivo [51]. The rising blood levels of LBP and sCD14 may serve as a compensatory mechanism to prevent excessive TLR4 stimulation and to limit systemic inflammation in patients with ischemic stroke [61]. In the study by Klimiec et al., the source of circulating LPS could not be determined, and there was no difference in serum levels between patients with and without infection, which raised the possibility of bacterial translocation as a contributing factor. This suggests that LPS might enter the bloodstream from the gut rather than from an overt infection [61]. In our study, the serum levels of LBP and sCD-14 were significantly higher in the unfavorable outcome group among aSAH patients without in-hospital infection.

Based on this, it can be stated that in our current study, although the molecules examined are well-documented serum markers of gut permeability, they do not clearly prove that the appearance of LBP in the systemic circulation is definitely a product of microorganisms related to the gut microbiome. Nevertheless, the body’s microbiome systems (e.g., lungs, gonads, etc.) may also be affected by the pathophysiological stress caused by aSAH. Therefore, endotoxins could be released from these microbial compartments as well. Thus, our results primarily indicate that while the precise origin of the LBP and sCD-14 appearing in the circulation and reaching significantly higher levels in the unfavorable outcome group cannot be confirmed, one potential source could be the human gut microbiome, whose endotoxins may enter the systemic circulation during the early phase of aSAH due to impaired gut permeability.

Our current study also found that the serum levels of LBP and sCD14 were significantly higher in the subgroup with DCI. Since DCI typically occurs between days 7 and 14 [64], the difference observed in our study between the DCI-positive and DCI-negative groups in the late phase regarding LBP and sCD-14 serum levels may indicate a correlation between these two markers and DCI. Our findings suggest a connection between DCI and elevated levels of such permeability markers. Importantly, patterns of certain cytokines observed in the sera of patients with aSAH are linked to the development of DCI [65]. Considering the previously outlined relationship between the LPS–LBP–sCD14 interaction in activating the inflammatory response [51,52], we hypothesize that the endotoxins entering the circulation during aSAH may play a direct role in the development of delayed cerebral ischemia (DCI) by initiating and sustaining inflammatory processes.

Our study has several limitations. The origin of the endotoxins could not be determined in our current study. We did not examine the composition of the gut microbiome, so we cannot confirm the presence of any dysbiosis. The markers we investigated are indirect indicators of changes in gut permeability, and their specificity cannot be measured against the specificity of direct assessments of intestinal permeability. The additional limitation is that we did not measure the amount of bacteria that may have entered the circulation through the leaky gut, nor the quantity of their genome. We cannot rule out the possibility that damage to the normal flora of other organs (e.g., the lungs) may also play a role in our results. The methodology of the current study does not allow for determining whether the differences observed in the markers for the examined endpoints are causative factors or consequences. Further research is required to address this question.

These limitations highlight the need for further research to establish a clearer understanding of gut health and its implications for conditions such as delayed cerebral ischemia. Future studies might consider including comprehensive microbiome analyses alongside direct permeability tests to enhance the specificity and relevance of findings related to gut health and systemic inflammation.

In conclusion, while the studied markers do not aid in the early prediction of DCI, LBP in the early phase (D1) is indicative of an unfavorable prognosis. Notably, both sCD-14 and LBP significantly differentiate between outcome groups in the infection-free cohort, even during the early phase. These findings highlight the potential of these biomarkers for early outcome stratification, though further studies are needed to clarify their causative role.

## 4. Materials and Methods

### 4.1. Study Design and Population

Institutional review board approval was obtained previously (IV/8468-1/2021/EKU). Written informed consent was obtained from patients or their legal representatives prior to their inclusion in the study. Patients diagnosed with aSAH at our institution from February 2021 to November 2023 were prospectively included in the study. Our study’s inclusion criteria were as follows: 1. age > 18 years old; 2. confirmed diagnosis of aSAH by non-contrast head CT, and established diagnosis of aneurysm by CTA or DSA; 3. diagnosis occurred within 24 h after index event. Patients with traumatic SAH, pregnancy, hospital admission later than 24 h after ictus, no aneurysm treatment, bleeding from arteriovenous malformation, absence of a signed consent form, underlying systemic diseases (malignancies, liver and/or renal insufficiency, and chronic lung disease, inflammatory bowel disease or any known chronic gastrointestinal diseases), chronic infection or signs of any acute infection on admission were excluded. Patients who experienced rerupture after the ictus or deteriorated during treatment were also excluded from the study. For eligible patients, their medical records, including age, sex, risk factors (hypertension, diabetes, smoking status), blood pressure and laboratory measurements on admission, baseline WFNS and mFisher score, interventions performed during intensive care (mechanical ventilation, extraventricular drain, lumbar drain, etc.), occurrence and type of infection, occurrence of DCI and clinical outcomes (modified Rankin Scale, mRS) were reviewed and recorded. All patients were followed up through phone calls or personal visits, mRS at 3 months was recorded to assess the clinical outcome, the favorable outcome was defined as an mRS of 0–3 at the follow-up [66]. The primary outcome was the correlation between serum levels of gut permeability markers (FABP-I, LBP, sCD-14) and the 3-month clinical outcomes in aSAH patients. The secondary outcomes include the association of these markers with delayed cerebral ischemia (DCI) in the early and late phases of aSAH and the potential differentiation between patients with favorable and unfavorable outcomes based on the absence of in-hospital infections. Comparisons between healthy controls and aSAH patients were conducted to establish baseline differences. The outcome data were assessed by independent, trained, blinded professionals, not associated with the study, 3 months (±days) following the aSAH event. All patients received nimodipine 6 times 60 mg per os from the first day for vasospasm prevention. Delayed cerebral ischemia (DCI) was identified based on previously established criteria [67]. The diagnosis of DCI was made only after thoroughly ruling out other potential causes and required the consensus of at least two neurointensivists. We considered a patient to be DCI-positive if clinical deterioration and/or ischemic lesions, confirmed by CT/MR, occurred within 6 weeks of the admission and could not be explained by other causes. Large vessel vasospasm, in the absence of clinical symptoms or a new ischemic lesion, was not considered as DCI [67]. The criteria for defining systemic and central nervous system infection included the presence of infection symptoms with fever (>38 °C), elevated levels of C-reactive protein (rising CRP level after an initial peak) and/or procalcitonin (>0.5 ng/mL), combined with a positive diagnostic test, such as a chest X-ray, CSF, and/or blood culture or urine analysis. The control group consisted of patients who have a cerebral aneurysm confirmed by CT/MR imaging, but whose treatment was not indicated due to low risk, or who declined aneurysm treatment. In these patients, we conducted serum sampling and verified the exclusion criteria consistent with the patient population. Ongoing infections, which could affect the measured marker levels, were ruled out through laboratory tests.

### 4.2. Sampling and Analysis of Markers

Arterial blood samples were collected from all eligible patients on the 1st day (D1) after ictus and 9th day (D9) following the ictus. These samples were centrifuged within 30 min, and the serum supernatant was stored at −80 °C until analysis. Serum levels of sCD14, LBP, and FABP-I were determined using Human CD14, LBP, and FABP2/I-FABP DuoSet ELISA kits (Bio-Techne, Minneapolis, MN, USA) according to the manufacturer’s protocols. The reaction was developed with TMB and measured at 450 nm using an iEMS MF microphotometer (Thermo Labsystem, Beverly, MA, USA).

### 4.3. Statistical Analysis

The data were analyzed using the SPSS 25.0 (SPSS Statistics v22.0; IBM Corp., Armonk, NY, USA). Graphs were drawn using the GraphPad Prism 10.0 (GraphPad Software, Inc., Boston, MA, USA). Qualitative variables were represented as frequencies (percentages), while normally and non-normally distributed continuous variables were presented as means (standard deviations, SD) and medians (interquartile ranges), respectively. Comparisons of different variables between the two groups were conducted using the Chi-square test or Fisher’s exact test for qualitative data, and the Mann–Whitney U test or independent *t*-test for quantitative data. The Kruskal–Wallis test was utilized for comparing quantitative data across multiple groups. The Spearman correlation coefficient was used to calculate the bivariate correlation between serum FABP-I, LBP, and sCD-14 levels.

## 5. Conclusions

The early changes in the serum levels of gut permeability markers compared to the control suggest the possibility of gut permeability dysfunction already in the early phase of aSAH. In patients without infection, the difference in LBP and sCD-14 levels observed between the outcome groups cannot be clearly explained, but in the absence of confirmed infection, the possibility of endotoxin release from the body’s microbial systems arises, which may contribute to neuroinflammation and influence the outcome.

## Figures and Tables

**Table 1:** Demographic and clinical characteristics of aSAH population.

Variable	Total (*n* = 177)	Favorable (*n* = 94)	Unfavorable (*n* = 83)	*p*-Value
Age (mean ± SD)	57, 8 ± 12	54 ± 10	62 ± 12	<0.001
Female, *n* (%)	128 (72)	65 (69)	63 (76)	0.202
Hypertension, *n* (%)	90 (51)	44 (47)	46 (55)	0.180
Diabetes, *n* (%)	18 (10)	8 (9)	10 (12)	0.452
Smoking, *n* (%)	56 (32)	35 (37)	21 (25)	0.047
WFNS, median (IQR)	2 (1–4)	1 (1–2)	4 (2–5)	<0.001
mFisher score, median (IQR)	3 (2–3.5)	2 (2–3)	3 (3–4)	<0.001
Loss of consciousness during ictus, *n* (%)	66 (37)	27 (29)	38 (47)	0.01
Seizure during ictus, *n* (%)	18 (10)	6 (6)	12 (15)	0.031
Aneurysm location, *n* (%)				
internal carotid artery	13 (7)	6 (7)	7 (8)	
middle cerebral artery	50 (28)	24 (26)	26 (30)	
anterior communicating artery	56 (32)	29 (32)	27 (32)	
posterior communicating artery	19 (11)	13 (14)	6 (7)	
anterior cerebral artery	9 (5)	5 (6)	4 (5)	
vertebrobasilar	30 (17)	14 (15)	16 (18)	
Aneurysm multiplicity, *n* (%)	44 (25)	27	17	0.093
Blood pressure, systolic ^a^, median (IQR)	150 (131–171)	150 (131–170)	150 (131–180)	0.769
Blood pressure, diastolic ^a^, median (IQR)	85 (79–94)	89 (80–97)	84 (78–92)	0.107
C-reactive protein ^a^, mg/L, median (IQR)	15.5 (4–55)	8 (3–17)	49 (9–88)	<0.001
Creatinine ^a^, µmol/L, median (IQR)	60 (49–71)	59 (49–68)	65 (49–76)	0.052
White blood cell count ^a^, G/L, median (IQR)	10.8 (9–13)	10 (8–13)	12 (10–15)	0.016
Neutrophile-lymphocyte ratio ^a^, median (IQR)	5.8 (4–10)	4 (3–7)	7 (5–12)	<0.001
Lumbar drain, *n* (%)	91 (51)	49 (52)	42 (51)	0.479
Mechanical ventilation, *n* (%)	82 (46)	13 (14)	69 (83)	<0.001
Decompressive craniotomy, *n* (%)	17 (9.6)	1 (1)	16 (19)	<0.001
Extraventricular drainage, *n* (%)	81 (46)	17 (18)	64 (77)	<0.001
Delayed cerebral ischemia, *n* (%)	47 (27)	7 (7)	40 (49)	<0.001
Infection, *n* (%)	61 (34)	14 (15)	47 (57)	<0.001

SD, standard deviation, *n*, number, WFNS, World Federation of Neurological Societies, IQR, interquartile range, ^a^ on admission. We defined favorable outcomes as modified Rankin score (mRS) 0–3, while unfavorable outcomes were determined as mRS 4–6.

## Data Availability

The datasets used and/or analyzed during the current study are available from the corresponding author on reasonable request.

## Supplementary Materials

The following supporting information can be downloaded at: https://www.mdpi.com/article/10.3390/ijms26020485/s1.
