# Supplementary material for: Effects of Aneurysmal Subarachnoid Hemorrhage in Patients Without In-Hospital Infection on FABP-I, LBP, and sCD-14
Source: Int J Mol Sci. 2025 Jan 8;26(2):485. doi: 10.3390/ijms26020485 (PMC11764490; doi:10.3390/ijms26020485)
Supplement: Supplementary file 1 [file ijms-26-00485-s001.zip › File S2.pdf]

Suppmentary file S2. Exact value of measured markers.

Variable	Total (n=177)	Favorable (n=94)	Unfavorable (n=83)	*P*-value
FABP-I, D1, pg/mL, median (IQR)	410 (252-647)	357 (248-600)	411 (267-769)	0.364
LBP, D1, ng/mL, median (IQR)	23475 (16311-39612)	18798 (12618-30566)	28932 (21552-46908)	<0.001
sCD-14, D1, ng/mL, median (IQR)	3584 (2278-5492)	3461 (2170-5017)	4200 (2327-6057)	0.154
FABP-I, D9, pg/mL, median (IQR)	178 (107-281)	193 (119-303)	147 (75-223)	0.065
LBP, D9, ng/mL, median (IQR)	25500 (17028-41955)	21348 (14450-33132)	33849 (25538-46893)	<0.001
sCD-14, D9, ng/mL, median (IQR)	4102 (2360-6144)	3305 (2196-5426)	4625 (3059-6639)	<0.001
FABP-I, ctrl, pg/mL, median (IQR)	720 (436-929)			
LBP, ctrl, ng/mL, median (IQR)	7437 (5433-10302)			
sCD-14, ctrl, ng/mL, median (IQR)	1660 (1232-2494)			
