# Supplementary figures and images for: Effects of Aneurysmal Subarachnoid Hemorrhage in Patients Without In-Hospital Infection on FABP-I, LBP, and sCD-14
Source: Int J Mol Sci. 2025 Jan 8;26(2):485. doi: 10.3390/ijms26020485 (PMC11764490; doi:10.3390/ijms26020485)

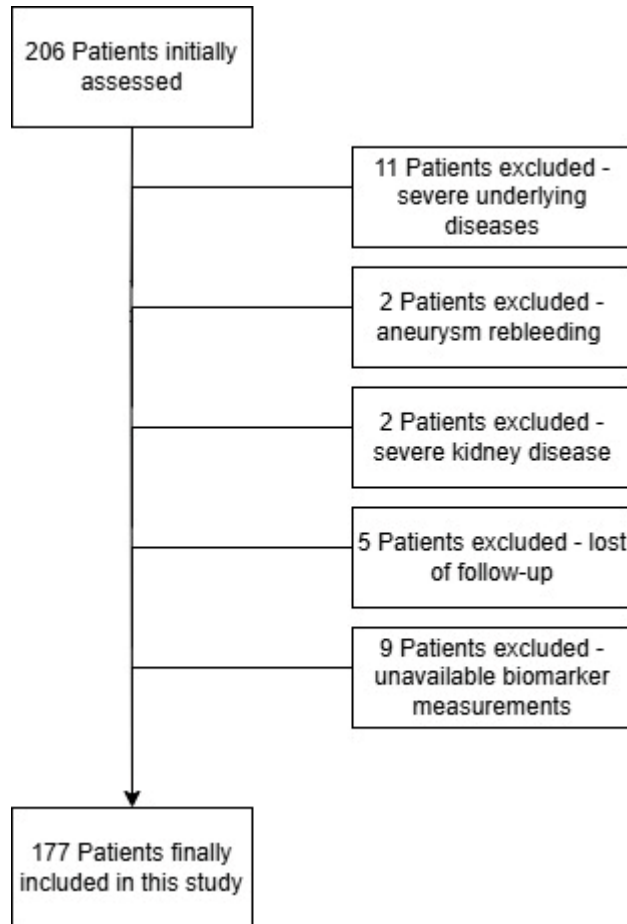

Supplement: Supplementary file 1 [file ijms-26-00485-s001.zip › File S1.pdf]
